# Interplay of Demographic Influences, Clinical Manifestations, and Longitudinal Profile of Laboratory Parameters in the Progression of SARS-CoV-2 Infection: Insights from the Saudi Population

**DOI:** 10.3390/microorganisms12051022

**Published:** 2024-05-18

**Authors:** Sarah Al-Hassinah, Sooad Al-Daihan, Mashael Alahmadi, Sara Alghamdi, Rawabi Almulhim, Dalia Obeid, Yaseen Arabi, Abdulrahman Alswaji, Marwh Aldriwesh, Majed Alghoribi

**Affiliations:** 1 Infectious Diseases Research Department, King Abdullah International Medical Research Center, Riyadh 11426, Saudi Arabia; s.alhassinah@gmail.com (S.A.-H.); alghamdisa13@mngha.med.sa (S.A.); arabi@mngha.med.sa (Y.A.); alswajiab@gmail.com (A.A.); aldriweshm@ksau-hs.edu.sa (M.A.); 2 Biochemistry Department, College of Science, King Saud University, Riyadh 11495, Saudi Arabia; sdaihan@ksu.edu.sa; 3 Research Office, Saudi National Institute of Health (SNIH), Riyadh 12382, Saudi Arabia; alahmadi.mashael@hotmail.com; 4 Infection Prevention and Control Department, King Abdulaziz Medical City, Riyadh 14611, Saudi Arabia; almulhemra@mngha.med.sa; 5 King Faisal Specialist Hospital and Research Center, Riyadh 11564, Saudi Arabia; obeiddx@gmail.com; 6 Intensive Care Department, King Abdulaziz Medical City (KAMC), Ministry of National Guard Health Affairs (MNGHA), Riyadh 11426, Saudi Arabia; 7 College of Medicine, King Saud Bin Abdulaziz University for Health Sciences, Riyadh 14611, Saudi Arabia; 8 Department of Clinical Laboratory Sciences, College of Applied Medical Sciences, King Saud Bin Abdulaziz University for Health Sciences, Riyadh 11481, Saudi Arabia; 9 Department of Basic Science, College of Science and Health Professions, King Saud Bin Abdulaziz University for Health Sciences, Riyadh 14611, Saudi Arabia

**Keywords:** SARS-CoV-2 infection, risk factors, age, gender, comorbidity, clinical manifestations, laboratory parameters, severity progression

## Abstract

Understanding the factors driving SARS-CoV-2 infection progression and severity is complex due to the dynamic nature of human physiology. Therefore, we aimed to explore the severity risk indicators of SARS-CoV-2 through demographic data, clinical manifestations, and the profile of laboratory parameters. The study included 175 patients either hospitalized at King Abdulaziz Medical City–Riyadh or placed in quarantine at designated hotels in Riyadh, Saudi Arabia, from June 2020 to April 2021. Hospitalized patients were followed up through the first week of admission. Demographic data, clinical presentations, and laboratory results were retrieved from electronic patient records. Our results revealed that older age (OR: 1.1, CI: [1.1–1.12]; *p* < 0.0001), male gender (OR: 2.26, CI: [1.0–5.1]; *p* = 0.047), and blood urea nitrogen level (OR: 2.56, CI: [1.07–6.12]; *p* = 0.034) were potential predictors of severity level. In conclusion, the study showed that apart from laboratory parameters, age and gender could potentially predict the severity of SARS-CoV-2 infection in the early stages. To our knowledge, this study is the first in Saudi Arabia to explore the longitudinal profile of laboratory parameters among risk factors, shedding light on SARS-CoV-2 infection progression parameters.

## 1. Introduction

The SARS-CoV-2 pandemic has changed global health prospects dramatically and has become a remarkable challenge globally [1]. SARS-CoV-2 infection demonstrates a broad range of severity, from cases with no symptoms to severe cases with fatal complications, which significantly increase the mortality rate worldwide [2]. Previous research has identified individual risk factors contributing to severe SARS-CoV-2 infection outcomes [3,4], but understanding the complex interactions among these factors is critical, yet remains limited. Demographic factors including age, gender, and comorbidities are well-recognized as determinants of SARS-CoV-2 infection outcomes [5,6,7]. In particular, elderly patients and patients with comorbidities have been consistently associated with an increased risk of infection severity and fatality [6,8,9]. However, the exact mechanisms through which these demographic factors interact with clinical symptoms and laboratory findings to influence infection severity and long-term implications are still complex and not fully understood [10,11,12,13,14].

Clinically, the symptoms of SARS-CoV-2 ranged based on infection severity, which demonstrates valuable insights into the progression of the disease [15,16]. The most commonly observed symptoms were respiratory symptoms, including cough and shortness of breath. Other clinical presentations, such as fatigue, loss of taste or smell, and gastrointestinal disturbances, have also been reported [2]. Clinical laboratory parameters, including complete blood count, inflammatory markers, organ function tests, and coagulation markers, were varied in SARS-CoV-2 infection cases and were used to reflect the physiological changes occurring within the body [17,18]. It is therefore crucial in following patients’ progress and predicting potential complications [18]. In this study, we explore the interplay between the severity of SARS-CoV-2 infection and progression parameters by investigating the demographic influences, clinical manifestations, and longitudinal profile of clinical laboratory parameters.

## 2. Materials and Methods

### 2.1. Study Design and Participants

The study was conducted at King Abdulaziz Medical City–Riyadh (KAMC) and King Abdullah International Medical Research Centre (KAIMRC), which are part of the Ministry of National Guard Health Affairs (MNGHA), Saudi Arabia. This cohort study included 175 patients with confirmed SARS-CoV-2 infection, either hospitalized at KAMC or placed in quarantine at assigned hotels in Riyadh, Saudi Arabia, from June 2020 until April 2021. Exclusion criteria included individuals younger than 18 years old, pregnant women, those testing negative for SARS-CoV-2 infection via real-time polymerase chain reaction (RT-PCR), patients who had immunocompromising conditions or who were receiving immunosuppressant medications, and patients with incomplete data records. Positive cases of SARS-CoV-2 were confirmed by testing nasopharyngeal swab samples, which were processed and analyzed in the Division of Molecular Virology, Department of Pathology and Laboratory Medicine at KAMC, Riyadh, for SARS-CoV-2 identification using RT-PCR. This was carried out using the BIOFIRE FILMARRAY multiplex PCR system (BioMerieux, Craponne, France). Written informed consent was obtained from the patients in English/Arabic, prior to enrollment in the study.

### 2.2. Data Collection and Laboratory Analysis

The patients’ demographic and clinical data were obtained on admission from the hospital’s information system database. Blood tests were conducted using standard laboratory methods comprised of complete blood count, activated partial thromboplastin time (APTT), prothrombin time (PT), the international normalized ratio (INR), alanine aminotransferase (ALT), bilirubin, C-reactive protein (CRP), aspartate aminotransferase (AST), glucose, blood urea nitrogen (BUN), lactate dehydrogenase (LDH), creatine kinase (CK), and creatinine (Cr).

### 2.3. Disease Severity Categorization and Comparative Analysis

Patients were stratified into three categories based on infectious disease progression: mild (quarantine, non-hospitalized), moderate (hospitalized, no-intensive care unit [ICU] admission), and severe (hospitalized, ICU-admitted), in accordance with the criteria of the World Health Organization [2]. Mild cases were characterized by the absence of pneumonia, whereas moderate cases presented with evidence of moderate pneumonia. Severe cases, on the contrary, were defined by meeting one or more of the following critical criteria: a respiratory rate of 30/min or more, an oxygen saturation level of less than 93%, a ratio of arterial partial oxygen pressure to inspiratory oxygen fraction (PaO2/FiO2) of less than 300 mmHg, the occurrence of respiratory failure necessitating mechanical ventilation, or the presence of shock or other organ failures that required intensive care support. Subsequently, we conducted comparative analyses of patient demographics, clinical symptoms, and the presence of comorbidities across these three groups. This was followed by a comparison of laboratory parameters between the moderate and severe groups. The latter were specifically chosen due to their hospital admission, allowing for a detailed follow-up. These follow-ups were conducted over a week at three distinct time points (second, third, and seventh days) post-admission.

### 2.4. Statistical Analysis

Descriptive statistics were utilized to present the data, including median values with minimum and maximum ranges (min–max) as well as both absolute and relative frequencies. For numerical data, statistical analyses were performed using the *t*-test or Wilcoxon rank sum test, contingent upon the assumptions of each test. A chi-square test was employed to assess the association between categorical variables. Univariable and multivariate logistic regression models were conducted to identify the factors contributing to the severity of SARS-CoV-2 infection and to compute the odds ratio for these associations. A binomial logistic regression model subsequently included variables demonstrating statistical significance in the univariate analysis. This model was applied with disease severity as the dependent variable to further elucidate the predictive factors of severe SARS-CoV-2 infection outcomes.

The threshold for statistical significance was established at *p* < 0.05. All the data analyses were conducted utilizing R version 4.1.2 [19] within the RStudio integrated development environment [20], ensuring robust and reliable statistical computations. All charts were generated using either GraphPad Prism 10 software or R version 4.1.2. within the RStudio integrated development environment.

### 2.5. Ethical Approval

This study received approval from the Institutional Review Board of the King Abdullah International Medical Research Center (KAIMRC), Riyadh, Saudi Arabia (IRB Approval Number: RC16/094/R). Prior to enrollment, informed consent was obtained from all participants, available in both English and Arabic, to ensure comprehensive understanding and voluntary participation.

## 3. Results

### 3.1. Demographics and Clinical Characteristics of Patients with SARS-CoV-2

**Figure 1:**
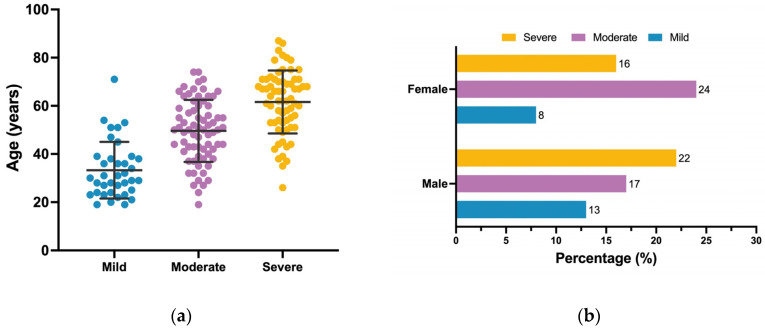
Distribution of age and gender across different severity levels of patients with SARS-CoV-2 infection. (**a**) Dot plots showcase the age distribution across the three severity levels: mild (blue), moderate (purple), and severe (yellow). The black lines within each plot represent the mean and standard deviation (SD). The width of the doted area at any given age value suggests the estimated data density. (**b**) A grouped bar chart presents the frequency distribution of gender (male and female) across the three severity levels. The severity levels are represented as mild (blue), moderate (purple), and severe (yellow). The length of each bar corresponds to the frequency count, also indicated numerically next to each bar.

The study cohort consisted of 175 confirmed SARS-CoV-2 cases, with 52.0% male patients (*n* = 91) and 48.0% female patients (*n* = 84). The median age was 47 years (range: 19–87) for male patients and 50 years (range: 19–83) for female patients. Among the patients, 67 cases (38.0%) were classified as severe, 71 (41.0%) as moderate, and 37 (21.0%) as mild SARS-CoV-2 infection. Mean ages, stratified by severity, were 33.3 ± 11.7 years for mild cases, 49.6 ± 12.9 years for moderate cases, and 61.6 ± 13.1 years for severe cases, as depicted in Figure 1a, which demonstrates an age-related escalation in severity (*p* < 0.0001). The results showed a significant association between the level of severity and gender (*p* = 0.0472), where male patients had a higher proportion in the severe group (22%), as demonstrated in Figure 1b. There was a significant variation in comorbidities status across different severity levels (*p* < 0.0001), with the severe SARS-CoV-2 infection group having the highest prevalence of comorbidities at 33.7%, followed by the moderate group at 29.7% and a distinct drop to 5.1% in the mild group. Diabetes was notably prevalent in the severe group (25.1%) compared to 14.9% in the moderate group and just 1.7% in the mild group, indicating a significant trend (*p* < 0.0001). Similarly, hypertension followed this trend, being significantly higher in the severe group (25.1%), in contrast to 12.6% in the moderate group and only 2.8% in the mild group. The prevalence of asthma and chronic cardiac conditions did not differ significantly across the patient groups. A comprehensive summary of the patients’ characteristics stratified by severity is presented in Table 1.

### 3.2. Symptom Presentation across Disease Severity Levels of SARS-CoV-2 Infection

In 175 patients, symptom patterns at admission varied significantly across different severity levels, showing several patterns, as summarized in Table 2. Shortness of breath (dyspnea) was observed in 11.0% of mild cases, increasing to 77.0% in moderate cases and further to 88.0% in severe cases (*p* < 0.001), which might potentially indicate its association with disease severity. Headaches were more common in the mild group at 51.0% but substantially less frequent in moderate (9.9%) and severe (7.6%) cases (*p* < 0.001), which suggested a reverse association with infectious disease severity. Interestingly, the symptom of loss/decrease in smell was highly prevalent in the mild cases at 51.0% but was almost absent in the moderate (1.4%) and severe cases (0%) (*p* < 0.001). Similarly, the loss/decrease in taste followed this trend, being reported in 46.0% of mild cases and plummeting to 2.8% in the moderate and 0.0% in the severe cases (*p* < 0.001). Moreover, muscle body aches were observed in the mild group at 41.0%, decreasing significantly to 7.0% in the moderate and further to 3.0% in the severe cases (*p* < 0.001). Tachypnea was more associated with severe cases at 12.0% compared to 2.8% in the moderate cases (*p* = 0.022). Chest pain exhibited a progressive increase from mild (5.4%) to moderate (5.6%) cases, peaking at 20.0% in severe cases (*p* = 0.023). Poor appetite oral intake was higher in the moderate cases at 14.0%, compared to a lower occurrence in the severe cases (7.6%) (*p* = 0.031).

### 3.3. Initial Laboratory Parameters and Their Association with Disease Severity

**Figure 2:**
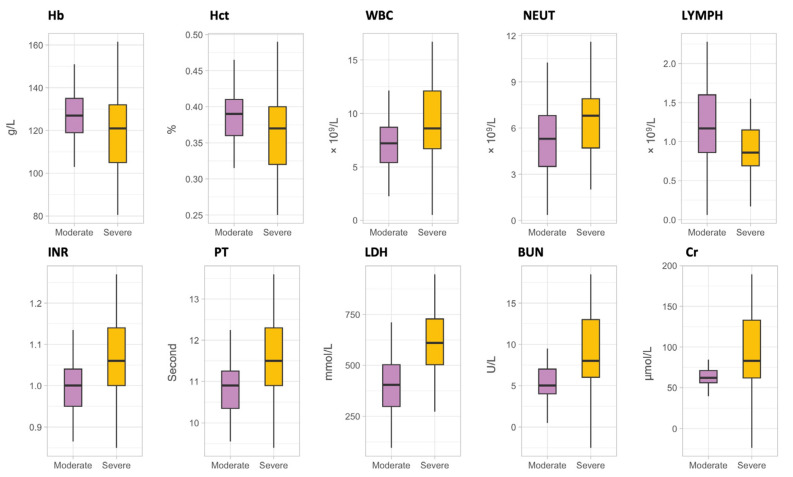
Clinical parameters demonstrating significant variations between moderate and severe patient groups of SARS-CoV-2 infection. In the boxplots, each box represents the interquartile range (IQR) with the median indicated by the central line. Whiskers extend to a maximum of 1.5 times the IQR from the box. Boxes are color-coded with moderate in lavender and severe in golden yellow. Abbreviation, Hemoglobin (Hb); Hematocrit (Hct); White Blood Cell Count (WBC); Neutrophils (NEUT); Lymphocytes (LYMPH); International Normalized Ratio (INR); Prothrombin Time (PT); Lactate Dehydrogenase (LDH); Blood Urea Nitrogen (BUN); Creatinine (Cr).

Laboratory parameters for the moderate and severe groups were evaluated upon initial admission, as presented in Figure 2. The median values with interquartile ranges (IQR) were presented for both the moderate (*n* = 71) and severe (*n* = 67) patient groups. Significant differences between these groups were observed, as detailed in Table 3. In the moderate group, the median hemoglobin level was 127 g/L (IQR: 119–135), which was significantly higher than the severe group’s median of 121 g/L (IQR: 105–132) (*p* = 0.027). Similarly, the hematocrit percentage was slightly higher in the moderate group, with a median value of 0.39% (IQR: 0.36–0.41) compared to 0.37% (IQR: 0.32–0.40) for the severe group (*p* = 0.040). Significant differences were also observed in white blood cell count, with the severe group showing a higher median count of 8.6 × 10^9^/L (IQR: 6.7–12.1) in comparison to 7.2 × 10^9^/L (IQR: 5.4–8.7) for the moderate group (*p* = 0.002). Regarding neutrophils and lymphocytes, the severe group’s median counts were 6.8 × 10^9^/L (IQR: 4.7–7.9) and 0.86 × 10^9^/L (IQR: 0.69–1.15), respectively, showing significant variation from the moderate group, which were 5.3 × 10^9^/L (IQR: 3.5–6.8) for neutrophils (*p* = 0.015) and 1.17 × 10^9^/L (IQR: 0.86–1.60) for lymphocytes (*p* = 0.022). Prothrombin time and international normalized ratio were significantly higher in the severe group, with medians of 11.50 s (IQR: 10.90–12.30) and 1.06 (IQR: 1.00–1.14), respectively, compared to the moderate group’s medians of 10.90 s (IQR: 10.35–11.25) for prothrombin time and 1.00 (IQR: 0.95–1.04) for international normalized ratio (both *p* < 0.001). Additionally, blood urea nitrogen, lactate dehydrogenase, and creatinine levels were significantly elevated in the severe group compared to the moderate group. Median values for these parameters in the severe group were 8 mmol/L (IQR: 6–13) for blood urea nitrogen, 610 U/L (IQR: 503–728) for lactate dehydrogenase, and 83 µmol/L (IQR: 62–133) for creatinine. In contrast, the values for the moderate group were 5 mmol/L (IQR: 4–7), 404 U/L (IQR: 298–503), and 62 µmol/L (IQR: 56–71) (*p* < 0.001 for blood urea nitrogen and creatinine, and *p* = 0.003 for lactate dehydrogenase). Other laboratory parameters, including platelets, monocytes, activated partial thromboplastin time, alanine aminotransferase, bilirubin, C-reactive protein, aspartate aminotransferase, glucose, and creatine kinase, did not exhibit significant differences between the moderate and severe groups (*p* > 0.05).

### 3.4. Longitudinal Laboratory Parameter Analysis

**Figure 3:**
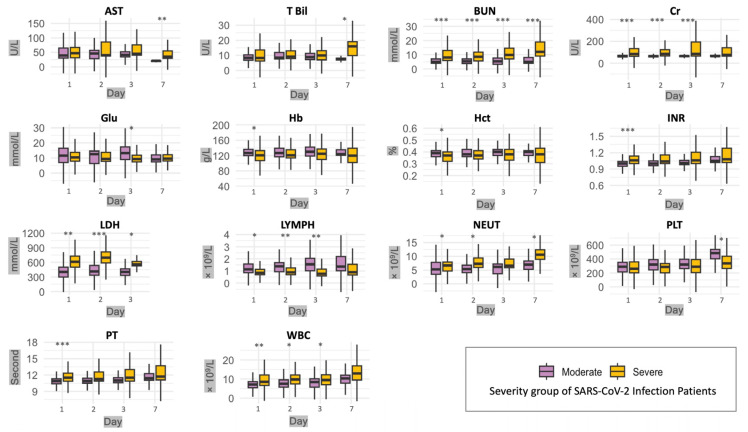
Comparative dynamics of laboratory parameters in the moderate and severe SARS-CoV-2 infection patients across a 7-day follow-up. Box plots display the interquartile ranges (IQR) of laboratory test values with moderate cases depicted in purple and severe cases in yellow. The median value for each group is indicated by the horizontal line within each box. Whiskers extend to a maximum of 1.5 times the IQR from the box. Data points outside this range are considered outliers and are represented by individual points. The laboratory tests consisted of aspartate aminotransferase (AST), total bilirubin (T Bil), blood urea nitrogen (BUN), creatinine (Cr), glucose (Glu), hemoglobin (Hb), hematocrit (Hct), international normalized ratio (INR), lactate dehydrogenase (LDH), lymphocyte count (LYMPH), a nutrition-related marker (NEUT), platelet count (PLT), prothrombin time (PT), and white blood cell count (WBC). The *x*-axis denotes the day of observation, while the *y*-axis shows the measured value of each test. Statistical significance between moderate and severe patient test results is indicated by asterisks (* = ≤0.05, ** = ≤0.01, and *** = ≤001).

Over seven days, the progression of key laboratory parameters in SARS-CoV-2 patients was tracked and categorized into moderate and severe groups. The laboratory parameters were initially assessed upon admission and subsequently re-evaluated at key time points on the second, third, and seventh days post-admission to document the patients’ clinical progression, as summarized in Figure 3 (for detailed values, refer to Table S1).

The second-day evaluation showed a significant continuous decrease in lymphocyte counts in the severe group (*p* = 0.009). In contrast, the severe group exhibited persistently high neutrophil counts (*p* = 0.013) and white blood cell counts (*p* = 0.021). Furthermore, the renal stress markers, blood urea nitrogen and creatinine, remained persistently elevated in the severe cases (*p* < 0.001 for blood urea nitrogen and *p* < 0.001 for creatinine). For the severe group, the median blood urea nitrogen level was 8.5 mmol/L and creatinine was 84 µmol/L. These values were significantly higher than those observed in the moderate group, where the median value was 5.4 mmol/L for blood urea nitrogen and 63 µmol/L for creatinine. Lactate dehydrogenase level was significantly elevated in the severe group, with a median of 696 U/L, compared to the median of 414 U/L in the moderate group (*p* < 0.001).

By the third day, the trends identified on the second day had further evolved, highlighting the ongoing progression of the disease in patients with severe SARS-CoV-2 infection. In the severe group, lymphocyte counts continued to decrease, with a significantly lower median (0.77 × 10^9^/L; *p* = 0.003), as compared to the moderate group. While not reaching statistical significance on this day (*p* = 0.1), neutrophil counts in the severe group remained elevated. Concerns persisted regarding renal markers; in the severe group, blood urea nitrogen and creatinine levels further increased to medians of 9.8 mmol/L (*p* < 0.001) and 84 µmol/L (*p* = 0.001), respectively. In the severe group, lactate dehydrogenase levels demonstrated a persistent elevation and remained significantly higher, with a median of 558 U/L, (*p* = 0.023), than levels in the moderate group.

By the seventh day, lymphocyte counts in severe cases showed a modest recovery (median 0.90 × 10^9^/L, IQR: 0.67–1.55), yet they remained reduced compared to moderate cases (median 1.37 × 10^9^/L, IQR: 1.02–2.19). Neutrophil counts in severe cases (median 10.6 × 10^9^/L, IQR: 8.9–12.4) continued to be substantially higher and statistically significant (*p* = 0.019) compared to those in moderate cases (median 7.0 × 10^9^/L, IQR: 5.3–8.3). Renal markers remained concerning in severe cases, exhibiting elevated blood urea nitrogen (median 12 mmol/L, IQR: 9–19, *p* < 0.001) and creatinine (median 75 µmol/L, IQR: 63–141, *p* = 0.15) levels. Platelet counts notably diverged, with severe cases showing lower counts (median 337 × 10^9^/L, IQR: 266–440) compared to the moderate cases (median 485 × 10^9^/L, IQR: 403–538, *p* = 0.017). On the seventh day, a liver function test revealed notably higher bilirubin levels in the severe group (median 16.0 µmol/L, IQR: 9.8–19.1). Similarly, aspartate aminotransferase levels were elevated in severe cases (median 35 U/L, IQR: 29–55) in comparison to moderate cases (median 21 U/L, IQR: 19–21, *p* = 0.006).

### 3.5. Multivariate Logistic Regression Models for Predicting Disease Severity

To evaluate the possibility of predicting the severity level of SARS-CoV-2 infection using early clinical characteristics and parameters, we included all variables that showed significance at admission in the univariate analysis in the multivariate logistic regression model. The model comprising 15 predictors demonstrated significant predictive power for SARS-CoV-2 infection severity, even in the absence of individually significant univariate predictors, as delineated in Table S2. Two additional models were developed to understand the significance of univariate risk factors. Predictors were categorized into two groups: demographic factors, summarized in Table 4, and clinical laboratory parameters on the first day of admission, summarized in Table 5. A logistic regression model incorporating the patients’ demographics and comorbidities was utilized to predict severity. The results indicated a lower risk of disease severity for female patients compared to male patients, with an adjusted odds ratio (aOR) of 0.42 (95% CI: 0.19–0.98). Additionally, age was a significant factor, with an increased severity risk observed in elderly patients (aOR = 1.1(1.1–1.12)). The multivariate model significantly predicted patients’ infection severity based on their demographic data (*p* < 0.0001). In the clinical laboratory tests model, no significant univariate predictors were identified, except for elevated urea levels, which notably indicated the highest risk in predicting disease severity (aOR = 2.56, 95% CI: 1.07–6.1). Furthermore, after adjusting for age, the multivariate model remained significant (*p* < 0.0001). However, individual predictors lacked significance in the univariate analysis, likely due to limitations such as sample size, and their combination within a multivariate model significantly enhanced predictive accuracy for disease severity.

## 4. Discussion

This study’s findings provide a broad examination of the demographics, comorbidities, clinical symptoms, and longitudinal profile of laboratory parameters of patients with SARS-CoV-2 at KAMC in Riyadh, Saudi Arabia, and highlight the risk factors of disease severity and progression. Identifying these indicators may help provide appropriate medical interventions and improvements in clinical care to enhance outcomes for SARS-CoV-2 patients.

Among 175 patients, there was an increase in the mean age among different levels of disease severity, with 33.3 ± 11.7 for mild cases, 49.6 ± 12.9 for moderate cases, and 61.6 ± 13.1 for severe cases, similar to the findings of studies performed in the United States, Italy, and Saudi Arabia [21,22,23]. This study highlights the critical role of age in disease severity, which aligns with aging characterization as a degenerative process, where accumulated damage results in cellular dysfunction and tissue and organ failure, ultimately leading to mortality [24]. The vulnerability of elderly people to severe respiratory infections and adverse outcomes is significantly increased by the chronic pro-inflammatory state of the aging immune system, which impairs immune responses [25,26], and the high prevalence of age-related comorbidities [27,28].

Our investigation reveals significant gender-based differences in SARS-CoV-2 infection severity, with the majority of the severe group being male (*p* = 0.0472). This aligns with prior studies on gender disparities in infectious diseases from different countries [29,30,31,32,33,34,35]. The evidence suggests that differences in immune responses between genders contribute to these variations in severity. Female patients generally exhibit more robust immune responses than male patients, potentially conferring a protective advantage against respiratory infections [32,36]. Behavioral factors, such as smoking habits, may further influence the observed gender disparities in disease severity [5]. Among this cohort, 68.5% (*n* = 120) had comorbidities, and they showed more association with severity level (*p* < 0.0001). However, this showed a higher percentage compared to earlier Saudi studies, which reported 20.1% (Alsofayan et al. [37]) and 29% (Khan et al. [38]), possibly due to the distribution of the sample size. Our findings revealed that the comorbidities most prevalent with severe infection were diabetes (*p* < 0.0001) and hypertension (*p* < 0.0001) [39]. These findings are consistent with earlier studies [21,22,23,37,38].

Symptom presentation varied significantly across severity levels. Notably, dyspnea was more common in severe cases. This observation is consistent with prior research findings identifying respiratory compromise as a hallmark of severe SARS-CoV-2 infection [16,40]. Such respiratory involvement is frequently associated with pathophysiological lung alterations, including alveolar damage and inflammation, culminating in compromised gas exchange [41,42]. In contrast, sensory symptoms such as anosmia and ageusia predominantly characterized milder cases, as reported in various studies [43,44]. Previous studies have suggested that anosmia can be a distinctive indicator of SARS-CoV-2 infection, even without other symptoms, such as rhinorrhea or nasal congestion [44,45,46,47,48]. These symptoms likely result from the virus’s effects on neural pathways and sensory organs, indicating a broader spectrum of viral effects beyond the respiratory system [49,50].

The current study findings regarding the longitudinal profile of laboratory parameters, including hemoglobin levels, white blood count, and renal and hepatic function biomarkers, corroborate with the existing literature that underscores the importance of these biomarkers in identifying and tracking the progression and severity of SARS-CoV-2 infection [51,52]. Early indicators from these biomarkers pointed to inflammation, suppression of the adaptive immune response, kidney injury, and tissue damage [53]. As the disease session progresses, elevated neutrophils and white blood cells indicate ongoing inflammation, potentially leading to tissue damage [53,54]. A continuous decrease in lymphocyte counts indicates the ongoing suppression of the adaptive immune response [54]. Renal stress markers, including blood urea nitrogen and creatinine, were significantly elevated, indicating potential acute kidney injury, which aligns with previous studies [55,56]. Furthermore, lactate dehydrogenase, an enzyme released during tissue breakdown [57], exhibited a significant increase, mirroring findings from previous studies suggesting that elevation signals extensive cellular damage [58,59,60]. Toward the end of the first week of admission, laboratory parameters indicated prolonged coagulopathy, evidenced by a decrease in platelet counts and potential liver stress, as indicated by elevated bilirubin levels consistent with numerous reports in the literature [61,62,63]. This temporal progression aligns with earlier reports demonstrating that the severity and outcome of the disease are influenced by multi-organ dysfunction, which highlights the complex nature of the disease [64,65]. This observation concurs with previous studies that emphasized the critical role of these biomarkers in predicting disease severity within the context of SARS-CoV-2 infection and other infectious diseases [64,66,67,68].

The multivariate logistic regression analysis highlighted the critical need to understand the interaction between patient characteristics and early biomarker detection in assessing SARS-CoV-2 infection severity and outcomes. These results build on and extend the findings of previous studies [69,70,71]. In our model, individual clinical and demographic factors did not demonstrate significant predictive power in the univariate analysis. However, they collectively exhibited a considerable ability to predict disease severity in the model, which aligns with earlier reported research in building models to assess severity in the early stage [69,70,71]. These findings reflect the accumulative effect of individual characteristics on disease presentation and severity level [69,70,71,72]. For instance, age has consistently been identified as a significant predictor in various studies, underscoring its multifaceted impact on the immune system and organ function [27,73,74,75,76]. The prevalence of age-related comorbidities has been shown to exacerbate this vulnerability, increasing the risk of adverse outcomes and mortality, a trend evident in SARS-CoV-2 infection and other infectious diseases [27]. Moreover, recent studies have highlighted gender-based variations in disease susceptibility and response [6]. These observations align with previous findings emphasizing gender-specific disparities in susceptibility to and outcomes for various infectious diseases [30,32,36]. Notably, elevated urea levels in the clinical laboratory tests model were significant, indicating the importance of renal function in SARS-CoV-2 infection severity [55,56]. The significant global *p*-value of both models supports the use of comprehensive models that integrate various clinical and demographic parameters for risk stratification in SARS-CoV-2 infection. These statistically significant models reflect the complexity of SARS-CoV-2 and the interplay between different factors in shaping the progression of the disease course and outcome. Nevertheless, limitations include a small sample size and the need for validation.

## 5. Conclusions

The study demonstrated that apart from laboratory parameters, age and gender reflect the risk of SARS-CoV-2 infection in the early stages. However, the longitudinal profile of laboratory parameters showed that some risk indicators presented later in the disease progression session. This emphasizes the need for further investigation to broaden these insights by incorporating genetic, socioeconomic, and behavioral variables. Such a comprehensive approach has the potential to improve the precision of predicting the level of SARS-CoV-2 infection severity in the early stages and impact patient care management.

## Figures and Tables

**Table 1:** Demographics and clinical characteristics of patients with SARS-CoV-2 infection stratified by the severity level.

	Patents’ Group, Total *n* = 175			*p*-Value ^1^
	Mild, 37 (21%)	Moderate, 71 (41%)	Severe, 67 (38%)	
**Age, Mean** (**SD**)	33.3 (11.7)	49.6 (12.9)	61.6 (13.1)	<0.0001 ^2^
**Gender**				
Female	14 (8.0%)	42 (24.0%)	28 (16.0%)	0.0472
Male	23 (13.1%)	29 (16.6%)	39 (22.3%)	
**Medically Free**				
No	9 (5.1%)	52 (29.7%)	59 (33.7%)	<0.0001
Yes	28 (16.0%)	19 (10.9%)	8 (4.6%)	
**Diabetes**				
No	34 (19.4%)	45 (25.7%)	23 (13.1%)	<0.0001
Yes	3 (1.7%)	26 (14.9%)	44 (25.1%)	
**Hypertension**				
No	32 (18.3%)	49 (28.0%)	23 (13.1%)	<0.0001
Yes	5 (2.8%)	2 2(12.6%)	44 (25.1%)	
**Chronic Cardia**				
No	36 (20.6%)	65 (37.1%)	56 (32.0%)	0.071
Yes	1 (0.57%)	6 (3.4%)	11 (6.3%)	
**Asthma**				
No	32 (18.3%)	60 (34.4%)	61 (34.9%)	0.50
Yes	5 (2.9%)	11 (6.3%)	6 (3.4%)	

^1^ Pearson’s chi-squared test for categorical variables, ^2^ *t*-test and F-test for numerical variables, Bold in 1st column indicates the main categories of the variables being compared.

**Table 2:** Symptom prevalence across different severity levels of patients with SARS-CoV-2 infection.

Symptom	Patients’ Group, Total *n* = 175			*p*-Value ^1^
	Mild, 37 (18%)	Moderate, 71 (34%)	Severe, 67 (33%)	
Fever	25 (68%)	47 (66%)	47 (71%)	0.8
Cough	9 (24%)	50 (70%)	46 (70%)	<0.001
Sputum	0 (0%)	1 (1.4%)	0 (0%)	>0.9
Sore Throat	2 (5.4%)	2 (2.8%)	1 (1.5%)	0.4
Runny Nose	9 (24%)	0 (0%)	0 (0%)	<0.001
Hemoptysis	0 (0%)	2 (2.8%)	0 (0%)	0.5
Dyspnea	4 (11%)	55 (77%)	58 (88%)	<0.001
Hypoxia	0 (0%)	0 (0%)	3 (4.5%)	0.11
Tachypnea	0 (0%)	2 (2.8%)	8 (12%)	0.022
Wheezing	2 (5.4%)	1 (1.4%)	0 (0%)	0.2
Chest Pain	2 (5.4%)	4 (5.6%)	13 (20%)	0.023
Headache	19 (51%)	7 (9.9%)	5 (7.6%)	<0.001
Abdominal Pain	2 (5.4%)	7 (9.9%)	3 (4.5%)	0.5
Nausea	5 (14%)	6 (8.5%)	5 (7.6%)	0.6
Vomiting	5 (14%)	14 (20%)	7 (11%)	0.3
Diarrhea	11 (30%)	13 (18%)	12 (18%)	0.3
Body ache	15 (41%)	5 (7.0%)	2 (3.0%)	<0.001
Fatigue	7 (19%)	14 (20%)	14 (21%)	>0.9
Tachycardia	0 (0%)	2 (2.8%)	0 (0%)	0.5
Poor appetite	0 (0%)	10 (14%)	5 (7.6%)	0.031
Confusion	0 (0%)	0 (0%)	1 (1.5%)	0.6
Loss/decrease smell	19 (51%)	1 (1.4%)	0 (0%)	<0.001
Loss/decrease taste	17 (46%)	2 (2.8%)	0 (0%)	<0.001

^1^ Pearson’s chi-squared test.

**Table 3:** Comparative laboratory measurements between moderate and severe groups of SARS-CoV-2 infection on the first day of admission.

Lab Measurement	N	Moderate, (*n* = 71) ^1^	Severe, (*n* = 67) ^1^	*p*-Value ^2^
Hemoglobin (g/L)	128	127 (119, 135)	121 (105, 132)	0.027
Hematocrit (%)	127	0.39 (0.36, 0.41)	0.37 (0.32, 0.40)	0.040
White Blood Cells (×10^9^/L)	126	7.2 (5.4, 8.7)	8.6 (6.7, 12.1)	0.002
Platelets (×10^9^/L)	124	292 (213, 351)	263 (204, 357)	0.5
Neutrophils (×10^9^/L)	95	5.3 (3.5, 6.8)	6.8 (4.7, 7.9)	0.015
Lymphocytes (×10^9^/L)	95	1.17 (0.86, 1.60)	0.86 (0.69, 1.15)	0.022
Monocytes (×10^9^/L)	95	0.43 (0.27, 0.57)	0.47 (0.30, 0.67)	0.8
Activated Partial Thromboplastin Time (Second)	89	28.0 (25.8, 30.2)	26.8 (25.1, 30.3)	0.5
Prothrombin Time (Second)	88	10.90 (10.35, 11.25)	11.50 (10.90, 12.30)	<0.001
The International Normalized Ratio	88	1.00 (0.95, 1.04)	1.06 (1.00, 1.14)	<0.001
Alanine Aminotransferase (U/L)	71	52 (28, 66)	38 (24, 53)	0.2
Bilirubin (umol/L)	68	8.2 (6.8, 10.3)	8.0 (6.1, 13.1)	0.8
C-Reactive Protein (mg/L)	31	38 (30, 93)	70 (35, 128)	0.6
Aspartate Aminotransferase (U/L)	71	41 (30, 66)	47 (31, 67)	0.6
Glucose (mmol/L)	105	11.5 (7.1, 16.1)	10.5 (7.9, 14.0)	0.6
Blood Urea Nitrogen (mmol/L)	125	5 (4, 7)	8 (6, 13)	<0.001
Lactate Dehydrogenase (U/L)	37	404 (298, 503)	610 (503, 728)	0.003
Creatine Kinase (U/L)	52	66 (55, 88)	86 (52, 144)	0.3
Creatinine (umol/L)	93	62 (56, 71)	83 (62, 133)	<0.001

^1^ Median (IQR); n (%). ^2^ Wilcoxon rank sum test.

**Table 4:** A logistic regression and adjusted odds ratio for patients demographics and comorbidities multivariate model.

Independent Variable	Severe Disease Risk aOR (95% CI)	Univariate *p*-Value	Global *p*-Value
**Age**	1.1 (1.1–1.12)	<0.0001	<0.0001
**Gender**		0.047	
Female	1		
Male	2.26 (1.0–5.1)		
**Comorbidity**		0.53	
No	1		
Yes	1.5 (0.43–5.0)		
**Diabetes**		0.49	
No	1		
Yes	1.39 (0.54–3.57)		
**Hypertension**		0.19	
No	1		
Yes	1.87 (0.73–4.78)		

Bold in 1st column indicates the main categories of the variables being compared.

**Table 5:** A logistic regression and adjusted odds ratio for the first day of admission clinical laboratory tests multivariate model.

Independent Variable	Severe Disease Risk aOR (95% CI)	Univariate *p*-Value	Global *p*-Value
Age	1.109 (0.996–1.236)	0.0603	<0.0001
Hemoglobin	1.032 (0.99–1.076)	0.1367	
White Blood Cells	2.03 (0.235–17.517)	0.5196	
Neutrophils	0.416 (0.035–5.016)	0.4899	
Lymphocytes	0.57 (0.032–10.138)	0.702	
Prothrombin Time	4.416 (0.813–23.991)	0.0855	
Blood Urea Nitrogen	2.561 (1.071–6.123)	0.0345	
Creatinine	1.016 (0.96–1.076)	0.5795	

## Data Availability

Data are contained within the article and supplementary materials.

## Supplementary Materials

The following supporting information can be downloaded at: https://www.mdpi.com/article/10.3390/microorganisms12051022/s1, Table S1: Seven-Day Laboratory Parameter Trends in SARS-CoV-2 infection: Distinguishing Moderate from Severe Cases; Table S2: Multivariate Analysis of Risk Factors for Severity in SARS-CoV-2 Patients.
