# Supplementary material for: Interplay of Demographic Influences, Clinical Manifestations, and Longitudinal Profile of Laboratory Parameters in the Progression of SARS-CoV-2 Infection: Insights from the Saudi Population
Source: Microorganisms. 2024 May 18;12(5):1022. doi: 10.3390/microorganisms12051022 (PMC11124088; doi:10.3390/microorganisms12051022)
Supplement: Supplementary file 1 [file microorganisms-12-01022-s001.zip › Table S1.pdf]

Table S1. Seven-Day Laboratory Parameter Trends in COVID-19: Distinguishing Moderate from Severe Cases.

	Day 1, n=137			Day 2, n= 94			Day 3, n=75			Day 7, n=49		
**Characteristic**	**Moderate, n = 71**	**Severe, n = 66**	**p-value**	**Moderate, n = 55**	**Severe, n = 39**	**p-value**	**Moderate, n = 35**	**Severe, n = 40**	**p-value**	**Moderate, n =11**	**Severe, n = 38**	**p-value**
Hemoglobin (g/L)	127(120-136)	121(107-133)	0.027	127(117-139)	121(114-135)	0.25	130(119-142)	125(110-137)	0.078	124(121-135)	120(102-139)	0.39
Hematocrit (%)	0.39(0.36-0.41)	0.37(0.32-0.40)	0.04	0.38(0.36-0.42)	0.37(0.34-0.41)	0.19	0.40(0.37-0.42)	0.38(0.33-0.42)	0.1	0.40(0.37-0.41)	0.38(0.31-0.43)	0.47
Whit Blood Cells (× 10^9/L)	7.2(5.5-8.7)	8.6(6.7-12.1)	0.002	7.6(5.8-9.6)	9.8(7.5-12.1)	0.021	8.5(5.8-10.1)	9.5(7.1-12.2)	0.036	10.3(7.9-12.0)	12.9(9.5-16.9)	0.069
Platelets (× 10^9/L)	291(216-352)	262(203-358)	0.42	321(245-391)	288(203-334)	0.1	321(264-396)	291(206-392)	0.35	485(403-538)	337(266-440)	0.017
Neutrophils (× 10^9/L)	5.3(3.5-6.8)	6.7(4.7-7.9)	0.025	5.4(4.1-6.8)	7.3(6.0-9.4)	0.013	6.2(3.7-7.2)	6.6(5.9-9.0)	0.1	7.0(5.3-8.3)	10.6(8.9-12.4)	0.019
Lymphocytes (× 10^9/L)	1.14(0.86-1.57)	0.85(0.69-1.14)	0.024	1.39(0.94-1.68)	0.89(0.69-1.26)	0.009	1.55(1.02-2.04)	0.77(0.59-1.16)	0.003	1.37(1.02-2.19)	0.90(0.67-1.55)	0.16
Prothrombin Time (seconds)	10.90(10.40-11.30)	11.50(10.88-12.33)	<0.001	10.90(10.50-11.40)	11.25(10.90-12.55)	0.053	11.0(10.6-11.5)	11.5(10.9-13.0)	0.051	11.40(11.05-12.25)	11.70(11.10-13.70)	0.48
The International Normalized Ratio	1.00(0.95-1.04)	1.06(1.00-1.14)	<0.001	1.00(0.96-1.05)	1.04(1.00-1.16)	0.082	1.01(0.98-1.06)	1.06(1.00-1.21)	0.066	1.05(1.02-1.13)	1.08(1.02-1.28)	0.48
Bilirubin (umol/L)	8.3(6.8-10.3)	8.2(6.3-13.6)	0.62	8.4(7.6-11.9)	9.1(7.8-13.0)	0.86	8.8(7.2-11.3)	9.9(7.0-13.1)	0.8	7.5(7.0-8.1)	16.0(9.8-19.1)	0.018
Aspartate Aminotransferase (U/L)	39(30-65)	47(31-67)	0.51	46(28-57)	40(37-86)	0.26	41(34-52)	45(40-76)	0.3	21(19-21)	35(29-55)	0.006
Glucose (mmol/L)	11.6(7.0-16.4)	10.4(7.9-13.8)	0.54	12.6(6.3-14.6)	9.4(7.8-13.8)	0.87	13.3(9.0-17.3)	9.3(7.4-11.9)	0.024	9.1(7.4-12.1)	9.6(8.0-12.2)	0.76
Blood Urea Nitrogen (mmol/L)	5(4-7)	8(6-13)	<0.001	5.4(3.8-7.0)	8.5(5.7-11.8)	<0.001	5.4(3.3-7.6)	9.8(7.0-14.6)	<0.001	5(4-8)	12(9-19)	<0.001
Lactate Dehydrogenase (U/L)	404(298-503)	610(503-728)	0.003	414(336-537)	696(587-815)	<0.001	404(333-468)	558(529-617)	0.023			>0.99

Creatinine (U/L)	62(56-71)	83(62-133)	<0.001	63(56-68)	84(65-123)	<0.001	64(58-68)	84(64-194)	0.001	63(60-71)	75(63-141)	0.15

Median (IQR)
Wilcoxon rank sum test; Wilcoxon rank sum exact test
