# Supplementary material for: Interplay of Demographic Influences, Clinical Manifestations, and Longitudinal Profile of Laboratory Parameters in the Progression of SARS-CoV-2 Infection: Insights from the Saudi Population
Source: Microorganisms. 2024 May 18;12(5):1022. doi: 10.3390/microorganisms12051022 (PMC11124088; doi:10.3390/microorganisms12051022)
Supplement: Supplementary file 1 [file microorganisms-12-01022-s001.zip › Table S2 .pdf]

Table S2. Multivariate Analysis of Risk Factors for Severity in COVID-19 Patients

Independent Variable	aOR	95% CL		Univariate p-value	Global p-value
Gender (REF=Female)	NA**	NA**	NA**	0.9489	0.03489*
Comrobdity (REF=No)	NA**	NA**	NA**	0.8901	
Diabetes (REF=No)	11.6	NA**	NA**	0.9857	
Hypertension (REF=No)	0.082	NA**	NA**	0.9848	
Age	2.018	0.002	NA**	0.8435	
Haemoglobin (g/L)	1.197	0.102	13.990	0.886	
Haematocrit (%)	NA**	NA**	NA**	0.8464	
White Blood Cell (× 10^9/L)	NA**	NA**	NA**	0.8536	
Nutrophils (× 10^9/L)	NA**	NA**	NA**	0.855	
Lymphocytes (× 10^9/L)	NA**	NA**	NA**	0.8372	
Prothrombin Time (seconds)	NA**	NA**	NA**	0.9253	
The International Normalized Ratio	NA**	NA**	NA**	0.9536	
Blood Urea Nitrogen (mmol/L)	0.565	NA**	NA**	0.983	
Lactate Dehydrogenase (U/L)	1.005	0.611	1.652	0.9856	
Creatinine (umol/L)	1.007	0.876	1.15	0.9173	

[*] Statistically significant value
[**] Not available, not enough sample size distributed to calculate ORs.
